# Computer-aided autotransplantation of teeth with 3D printed surgical guides and arch bar: a preliminary experience

**DOI:** 10.7717/peerj.5939

**Published:** 2018-11-22

**Authors:** Wei He, Kaiyue Tian, Xiaoyan Xie, Enbo Wang, Nianhui Cui

**Affiliations:** 1 Department of Oral and Maxillofacial Surgery, Peking University School and Hospital of Stomatology, Beijing, China; 2 Department of Oral and Maxillofacial Plastic and Traumatic Surgery, Capital Medical University School of Stomatology, Beijing, China; 3 Department of Oral and Maxillofacial Radiology, Peking University School and Hospital of Stomatology, Beijing, China

**Keywords:** Autotransplantation of teeth, Computer-aided surgical simulation, Surgical guide, Three-dimensional printing

## Abstract

**Background/Aim:**

Autotransplantation of teeth is a method to restore the missing teeth and computer-aided techniques have been applied in this field. The aim of this study was to describe a novel approach for computer-aided autotransplantation of teeth and to preliminarily assess its feasibility, accuracy, and stability.

**Methods:**

Eight wisdom teeth with complete root formation of eight adult patients were autotransplanted. Individual replicas of donor teeth with local splints, surgical templates, and arch bars were virtually designed and fabricated using three-dimensional printing, these were then applied in the autotransplantation surgeries. Clinical and radiological outcomes were observed, the extra-alveolar time, success rate, and 1-year survival rate were analyzed, and accuracy and stability of this approach were evaluated.

**Results:**

The extra-alveolar time of donor teeth were less than 3 min. The average follow-up duration was 2.00 ± 1.06 years. All autotransplanted teeth showed normal masticatory function. Ankylosis was found in one patient, and the overall success rate was 87.5%, whereas the 1-year survival rate was 100%. Linear differences between the designed and the immediate autotransplanted positions at crowns and apexes of the donor teeth were 1.43 ± 0.57 and 1.77 ± 0.67 mm, respectively. Linear differences between immediate and the stable positions at crowns and apexes of the donor teeth were 0.66 ± 0.36 and 0.67 ± 0.48 mm, respectively.

**Conclusion:**

The present study illustrated the feasibility, clinical satisfied accuracy, and stability of a novel approach for computer-aided autotransplantation of teeth. This new approach facilitated the surgical procedure and might be a viable and predictable method for autotransplantation of teeth.

## Introduction

As a valuable method for restoring missing teeth, autotransplantation of teeth has been used in clinical practice for over 60 years (Cross et al., 2013; Jang, Lee & Kim, 2013). The advantages and predicable outcomes of teeth autotransplantation have been well documented (Jang, Lee & Kim, 2013; Yoshino et al., 2012; Czochrowska et al., 2002; Chung et al., 2014). Studies on teeth autotransplantation have reported a success rate of 81–100% (Chung et al., 2014; Czochrowska et al., 2002; Yoshino et al., 2012) and a 5-year survival rate of approximately 90% (Yoshino et al., 2012).

Although several factors might affect the prognosis (Aoyama et al., 2012; Chung et al., 2014), autotransplantation of teeth is considered as a “technique-sensitive” procedure (Reich, 2008), or in other words, the surgical technique used plays an important role in autotransplantation of teeth. During the conventional process of autotransplantation of teeth, the donor teeth, as the only guide to prepare new sockets at recipient positions, needs to be extracted early and placed into new sockets for several times to check the fit of donor teeth into the new sockets. The prolonged extra-alveolar time and the damage to the periodontal ligament cells during multiple fitting attempts were both negative influencers of successful autotransplantation of teeth (Anssari Moin et al., 2016; Tsukiboshi, 2002).

More recently, computer-aided surgical simulation (CASS) has been used in the field of oral and maxillofacial surgery including autotransplantation of teeth (Cross et al., 2013; Jang, Lee & Kim, 2013; Lee & Kim, 2012). Rapid prototyping replicas based on cone-beam computed tomography (CBCT) data have been used as alternatives to donor teeth for preparation of new sockets, which have proven useful in reducing extra-alveolar time and injury to periodontal ligament cells (Jang, Lee & Kim, 2013; Lee & Kim, 2012; Van Der Meer et al., 2016; Verweij et al., 2017). In order to precisely transfer desired positions of the donor teeth to the clinic and to simplify the surgical procedure, clinicians tend to design certain types of individual surgical guides besides replicas (Shahbazian et al., 2013; Strbac et al., 2016; Van Der Meer et al., 2016). Several studies have assessed the accuracy of three-dimensional (3D) printed replicas of donor teeth (Khalil et al., 2016; Lee et al., 2015), but the accuracy of computer-aided autotransplantation of teeth alone has been evaluated by only a few in vitro studies (Anssari Moin et al., 2016, 2017). In addition, after transplantation of donor teeth to recipient sites, various position-maintaining methods have been used across different studies, including sutures, resin wire splints, and titanium screws (Bauss et al., 2002; Park et al., 2014; Zufia et al., 2017). To our knowledge, no study has reported a computer-aided fixation method yet.

The present study aimed to fabricate a series of novel surgical tools, including replicas, individual surgical templates, and individual arch bars, by using computer-aided design and 3D printing techniques and to evaluate the feasibility, accuracy, and stability of this new approach.

## Patients and Methods

### Patients

The present study was conducted in accordance with the guidelines of the Declaration of Helsinki and was approved by the institutional review board of the Peking University School and Hospital of Stomatology (PKUSSIRB-201734037). All participants provided written informed consent before participation.

In total, eight patients (four men, four women, average age: 26.88 ± 2.64 years) who needed restoration of one of their molars were enrolled in the present study. All of them fulfilled the following enrollment criteria: (i) aged 18–40 years; (ii) in good health condition and had no contraindications for oral surgery; (iii) the third molar could be used as a donor tooth; (iv) sufficient bone in the recipient position; (v) good oral hygiene; and (vi) showed satisfactory compliance to cooperate with treatment. All roots of donor teeth in this study were fully developed. The clinical characteristics of studied patients are listed in Table 1.

**Table 1:** Patient characteristics and clinical outcomes.

No	Gender	Age (years)	Donor tooth	Recipient site	Extra-alveolar time	Follow-up duration (years)	Time-point of stability (month)	Mobility
1	F	32	28	36	Immediate	4	6	No
2	M	27	28	26	Immediate	3	3	<1 mm
3	M	26	18	46	Immediate	2	3	No
4	M	28	48	46	Immediate	2	6	No
5	F	23	28	26	2 min 50 s	2	3	<1 mm
6	F	25	48	46	Immediate	1	3	No
7	F	26	38	36	2 min 40 s	1	3	No
8	M	28	48	46	Immediate	1	3	No

### Computer-aided surgery

One female patient whose wisdom tooth was transplanted to the first molar on the right side of the mandible was taken as an example to illustrate this novel clinical approach as follows.

The patients were scanned by the CBCT (NewTom VG; Quantitative Radiology, Verona, Italy) with the same scanning parameters (field of view: 8 × 12 cm, 110 kVp, 18 s, voxel size: 0.15 mm). The mesiodistal diameter as well as shape and length of the roots and root canals of the donor tooth were observed and recorded. A plaster cast of the mandibular dentition was fabricated for the patient, and subsequently, CBCT scans of the plaster cast were taken (CBCT 2). All CBCT data were stored in the DICOM (Digital Imaging and Communications in Medicine) format and imported into the mimics 16.0 (Materialise, Leuven, Belgium) software to generate a 3D digital jaw model named jaw 1 and a dentition model.

**Figure 1:**
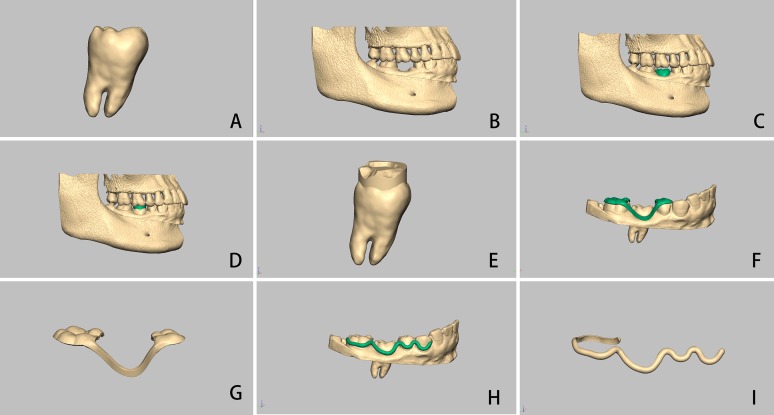
Computer-aided autotransplantation of tooth and virtual design of a series of novel surgical guides. (A) The donor tooth was isolated from CBCT data, (B) digital dentition was registered onto the digital jaw, (C) the digital donor tooth was transplanted onto the recipient site, (D and E) a local surgical split was generated between occlusal surfaces of the donor tooth and its opposite tooth and attached to the donor tooth, (F and G) individual surgical template was supported by adjacent teeth with a connecting bar, (H and I) virtually designed arch bar.

In the mimics software, the donor tooth was separated according to a modified method reported in a previously published study, and its corresponding 3D model was created (Fig. 1A) (Moin et al., 2013). The digital dentition model was registered on its corresponding digital jaw model such that the gingival margin could be recognized in the digital jaw model (Fig. 1B). Subsequently, the 3D model of the donor tooth was transplanted to the recipient site, and its location was finely adjusted to avoid any contact with its opposite tooth and to ensure that there was approximately one mm of distance between the occlusal surface of the donor tooth and its opposite teeth (Fig. 1C). Finally, the 3D model of the donor tooth in the desired position, the jaw (named as jaw 1), and the registered dentition were stored in an STL file, ready for the next step.

### Virtual design of surgical guides

The FreeForm Modeling Plus software (version 11; Geo Magics SensAble Group, Wilmington, MA, USA) and the Phantom Desktop arm (Geo Magics SensAble Group, Wilmington, MA, USA) system were used to virtually design the local surgical splint, individual surgical template, and arch bar.

The local surgical splint was generated between occlusal surfaces of the autotransplanted tooth and the opposite tooth. The splint was used to locate position of the replica of the donor tooth; hence, it was attached to the digital donor tooth (Figs. 1D and 1E).

Moreover, an individual surgical template was virtually designed to locate the position of the autotransplanted tooth during surgery. It had two ends placed on both adjacent teeth along with a connecting bar on the buccal surface of the autotransplanted tooth. The connecting bar of such a template has a “surface-to-surface” pattern contact with the donor tooth (Figs. 1F and 1G) (He et al., 2015a).

An individual arch bar was generated on the buccal aspect of the dentition from the first premolar to the second molar, including the donor tooth, in order to fix the donor tooth in the desired position after transplantation. The arch bar was shaped along the gingival edge but not compressing the gingiva (He et al., 2015a). The end of the arch bar was extended onto the lingual side of the second molar in order to increase surface area in contact as well as to improve stability. The inner surface of the arch bar had the same contact pattern as the connecting bar of the individual surgical template (Figs. 1H and 1I).

### Fabrication of surgical guides

**Figure 2:**
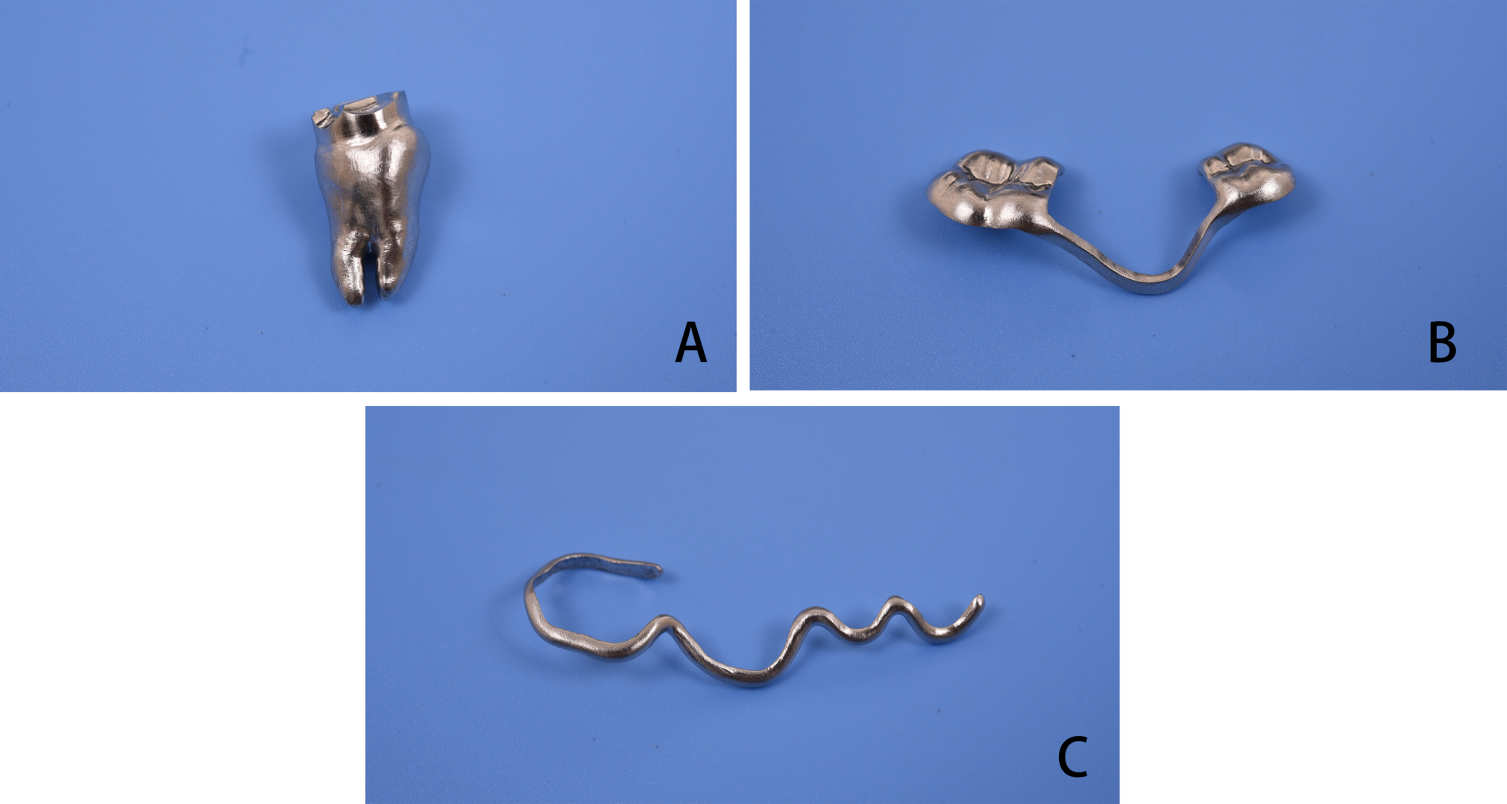
Three-dimensional print ing of surgical guides. (A) Replica of the donor tooth and the local split, (B) individual surgical template, (C) individual arch bar.

Data regarding the replica and the local surgical splint, individual surgical template, and arch bar in the STL format were imported into a 3D printing equipment (M280; Electro Optical System, Krailling, Germany) and prototyped using a cobalt–chrome (Co–Cr) alloy. Conventional polishing and sterilization processes were performed before their application in clinical surgery (Figs. 2A–2C).

### Surgical procedure

**Figure 3:**
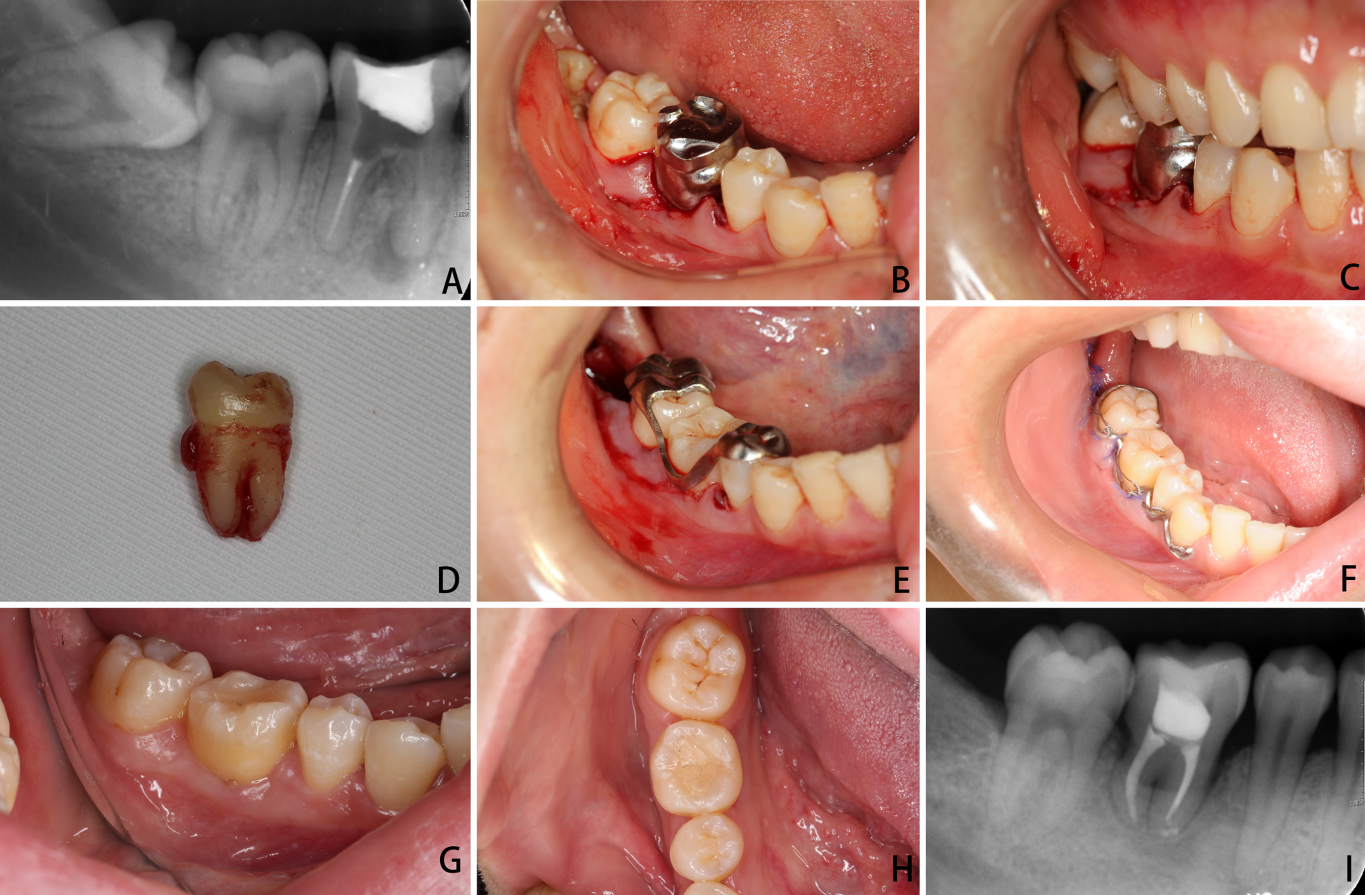
Autotransplantation surgery aided with individual guides. (A) Initial apical film of the first molar and the third molar that was selected to be the donor tooth, (B and C) the new socket at the recipient site was prepared under the guidance of a replica of the donor tooth and a local surgical split, (D) the donor tooth was gently extracted, (E) the donor tooth was immediately transplanted onto the recipient site, and the position of the autotransplanted tooth was checked using an individual surgical template, (F) 1 week after fixation with arch bar, (G and H) 1 year after the autotransplantation surgery: the gingiva was pink and firm and probing depth was ≤3 mm, (I) apical film of the autotransplanted tooth at 1 year.

The right mandibular first molar of the patient, used as the example here, was extracted before the autotransplantation surgery in order to avoid any consequent infection due to apical inflammation (Fig. 3A). After the guide, template, and arch bar were fabricated, the patient was called back for surgery under local anesthesia. During surgery, the new socket at the recipient position was prepared using a round bur. The replica with the local surgical splint was used as a guide before extraction of the donor tooth. It was a time-consuming process, and multiple fittings were attempted until the replica could fit into the new pocket and the splint could fit onto the occlusal surface of the opposite tooth (Figs. 3B and 3C). Subsequently, the donor tooth was gently extracted (Fig. 3D) and placed into the new socket, and the surgical template was used to check and confirm the desired position of the autotransplanted tooth (Fig. 3E). If the position of the donor tooth did not satisfactorily fit with the connecting part of the template (that is, the donor tooth did not fit into the designed position), the new socket was adjusted with additional preparation by using the guide of the extracted tooth. The extra-alveolar time was recorded. After the desired position of the donor tooth was achieved, the arch bar was ligated to the teeth with bifilar, 0.25-mm-diameter steel wires to fix the autotransplanted tooth (Fig. 3F).

### Follow-up

Cone-beam computed tomography scans were performed immediately after surgery, and recorded data were used to evaluate accuracy of the present approach (CBCT 3). Amoxicillin and metronidazole were prescribed for 1 week. Patients were followed up at 1 week, 2 weeks, 1 month, 3 months, 6 months, and 12 months after surgery, which were followed by subsequent annual visits. Clinical examinations were performed at each time point. Pulp vitality of the autotransplanted teeth were evaluated using an electrometric pulp tester at the 2-week time point, and the test results suggested that all donor teeth required root canal treatment (RCT). The arch bars were removed right after RCT was completed, usually at the 4-week time point. Periapical films were recorded at 3, 6, and 12 months and annually thereafter. To evaluate stability, CBCT scans of autotransplanted teeth were performed when the teeth achieved satisfactory function and stability (CBCT 4, Table 1).

The success criteria were modified from previous studies and included absence of root resorption and ankylosis, normal periodontal tissue, no deep pockets, and no excessive tooth mobility (horizontal movement >2 mm or any vertical movement) (Yoshino et al., 2012; Shahbazian et al., 2013; Yu et al., 2017). The survival criterion was that the transplanted tooth was still present at the last visit of the follow-up duration with or without fulfilling the aforementioned modified success criteria (Shahbazian et al., 2013; Yoshino et al., 2012).

### Accuracy and stability analysis

CBCT 3 and CBCT 4 data were separately imported into the mimics software, and the jaw with the autotransplanted tooth was segmented, reconstructed, and exported as an STL file, separately named as jaw 2 and jaw 3, respectively.

**Figure 4:**
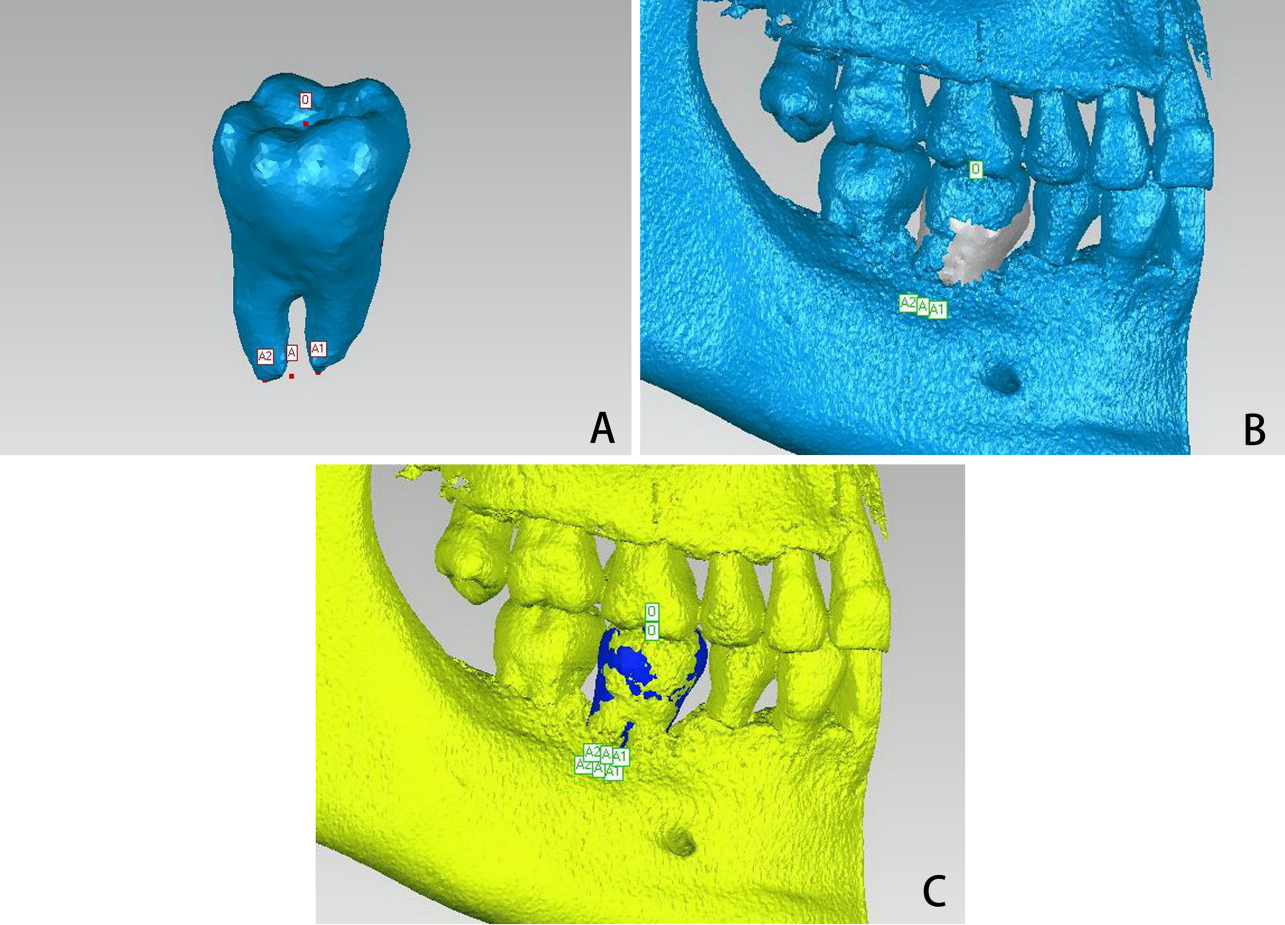
Analysis of accuracy and stability. (A) The bottom point of the central fossa on the designed donor tooth was labeled as point O, the middle point of the two apices was labeled as point A, and the coordinates were recorded, (B) jaw 2 was registered onto jaw 1, then, the designed donor tooth was registered onto the autotransplanted tooth, and new coordinates of the two points were recorded, (C) jaw 3 was registered onto jaw 2, the designed donor tooth was registered onto the stable autotransplanted tooth, and another new coordinates of the two points were recorded.

Subsequently, jaw 1 with the designed donor tooth and jaw 2 with the autotransplanted tooth were imported in the geomagic studio software (3D Systems, Rock Hill, SC, USA) to assess accuracy of the approach. The bottom point of the central fossa of the designed donor tooth on jaw 1 was labeled as point O to assess accuracy and stability of the crown. The apical point or the midpoint of the apical points of the designed donor tooth was labeled as point A to assess accuracy and stability of the apexes (Fig. 4A). The coordinates (X1, Y1, and Z1) of points O and A were recorded.

Next, jaw 2 was registered onto jaw 1 by selecting the common area of the bone (Fig. 4B). Subsequently, the designed donor tooth with these two points was registered onto the autotransplanted tooth, and the new coordinates (X2, Y2, and Z2) of points O and A were recorded as well. Differences between (X1, Y1, and Z1) and (X2, Y2, and Z2) would represent linear accuracy (Fig. 4B). To assess stability, jaw 3 with the stable autotransplanted tooth was imported into the geomagic studio software and registered onto jaw 2 by using the same aforementioned method. The designed donor tooth with the two points was registered onto the stable autotransplanted tooth, and the new coordinates (X3, Y3, and Z3) were recorded as well. Differences between (X2, Y2, and Z2) and (X3, Y3, and Z3) would represent linear stability (Fig. 4C).

The distances were measured for three times by the same examiner and the average values were used for statistical analysis.

### Statistical analysis

Descriptive statistics were performed using SPSS 20.0 (Chicago, IL, USA). Mean values and standard errors of mean of linear differences were presented.

## Results

The average follow-up duration was 2 ± 1.06 (range: 1–4) years. All autotransplanted teeth showed satisfactory stability by 3 or 6 months, with no mobility or mobility of <1 mm (horizontal movement, no vertical movement; Table 1). During the follow-up period, the teeth had normal masticatory function and no pathological symptoms. Depths of pockets ranged two to four mm. Gingivae were pink and firm with no bleeding on probing. No additional periodontal treatment was required. No continuous bone loss or root resorption was observed on radiographs. Ankylosis was found in one patient. The success rate was 87.5% (7/8), and the 1-year survival rate was 100% (8/8).

Linear differences of points O and A between the designed positions of donor teeth and the immediate positions of autotransplanted teeth at points O and A were 1.43 ± 0.57 and 1.77 ± 0.67 mm, respectively. Linear differences of points O and A between the immediate and the stable positions of autotransplanted teeth were 0.66 ± 0.36 and 0.67 ± 0.48 mm, respectively (Table 2).

**Table 2:** Linear accuracy and stability of the novel approach.

	*X* ± *s* (mm)	Max (mm)	Min (mm)
Linear accuracy			
Occlusal point	1.43 ± 0.57	2.08	0.69
Apical point	1.77 ± 0.67	2.32	0.72
Linear stability			
Occlusal point	0.66 ± 0.36	1.33	0.30
Apical point	0.67 ± 0.48	1.59	0.21

## Discussion

Autotransplantation of teeth has certain advantages over other methods of restoring missing teeth, such as potential for pulp revascularization, preservation of alveolar bone, no need for preparation of adjacent teeth, (Plakwicz, Wojtowicz & Czochrowska, 2013; Verweij et al., 2017) and more cost-effective in China (Yu et al., 2017). However, similar to other countries, autotransplantation of teeth is not very popular in China (Baviz, 2010; Jang, Lee & Kim, 2013). A study from Japan reported that the mean number of autotransplantation patients per clinic per year from 1990 to 2010 was just 1.4 (Yoshino et al., 2012). This might be because both surgeons and patients have doubts regarding the relatively complicated surgical procedure as well as the clinical outcomes, particularly long-term survival, and are hesitant to accept tooth autotransplantation, particularly in comparison with dental implants.

The CASS technique provided useful tools to assess donor teeth and recipient sites, to virtually perform the surgery on a computer, and to design surgical templates during the surgical planning stage, particularly when more than one teeth could be selected as the donor tooth. In the present study, a series of individual guides were designed, and the donor teeth were transplanted with the help of these guides. Results revealed that such novel surgical guides were useful and helped achieve satisfactory accuracy and stability. The local splints were extremely small and would not disturb a surgeon’s field of vision while preparing new sockets. After the donor teeth were extracted, six teeth could fit well into the new sockets, whereas additional preparation was required in two patients, and all donor teeth were transplanted within 3 min; thus, the extra-alveolar time was significantly reduced, and subsequently, damage of the periodontal ligaments was minimized (Verweij et al., 2017). This suggested that such guides could help surgeons decrease the challenges associated with autotransplantation.

Previous studies have reported various methods for fixation of transplanted teeth. However, this step is still not included in studies on computer-aided design. To maintain the transplanted teeth on the designed position after surgery, an individual arch bar was designed with a “surface-to-surface” contact pattern, and the donor and adjacent teeth were ligated with wires. The fixation method and duration for which it was maintained might influence clinical outcomes, wherein rigid fixation might exert negative effects on the autotransplanted teeth (Bauss et al., 2002; Chung et al., 2014). The arch bar used in this study was not a rigid fixation method, and the autotransplanted teeth were designed to be fit into infra-occlusal positions, which allowed the teeth to move in a limited range under a certain bite force during the initial stage after autotransplantation. Moreover, the physiological micromovements might enhance periodontal revascularization, and the mechanical stimulation might promote the proliferative activity of periodontal cells (Oortgiesen et al., 2012). Moreover, the arch bar could help the autotransplanted tooth to resist the movement caused by RCT and reduce any potential injury to the periodontal ligament.

Ankylosis was regarded as one of the criteria for failed autotransplantation. However, according to a previous study, ankylosis that is not associated with impairment of normal masticatory function should not be considered as treatment failure (Jang et al., 2016). Another two studies have reported unsuccessful outcomes of ankylosis of transplanted teeth with computer-aided techniques (Kim et al., 2005; Shahbazian et al., 2013). In addition, the space between the donor tooth root and the new prepared socket plays an important role in autotransplantation (Anssari Moin et al., 2016). Although the replicas have the same shape as the donor teeth, it was still difficult to design new pockets to ensure accurate 3D fitting with the shape of donor teeth roots. Moreover, mechanical stress between the sockets and the root surface of autotransplanted teeth might cause injury of periodontal ligaments and subsequently induce ankylosis (Hammarstrom, Blomlof & Lindskog, 1989; Jang et al., 2016). Custom-made drills and osteotomes might improve the accuracy and fit of the preparation before application of surgical robots in autotransplantation of teeth (Anssari Moin et al., 2016; Park et al., 2014). Furthermore, platelet-rich plasma or guided bone regeneration might have a promising use in autotransplantation of teeth to improve clinical outcomes (Gonzalez-Ocasio & Stevens, 2017; Yu et al., 2017).

Computer-aided surgical simulation opens the possibility to assess accuracy and stability of surgeries that require strict position control, such as orthognathic and dental implant surgeries (Behneke et al., 2012; He et al., 2015b). In the present study, linear deviation was calculated by using coordinate changes of label points. The results suggested that this novel approach could ensure and achieve clinically satisfactory accuracy and stability, which were within the clinically accepted range in the field of dental implants (Anssari Moin et al., 2017; Van Assche et al., 2012).

Despite the advantages of this novel approach, it still had a major disadvantage that it was a time-consuming procedure at the planning stage. It took approximately 4 h to perform the virtual surgery and design individual guides for each patient, apart from the time required for 3D printing; nevertheless, this approach could definitely reduce the time required for and increase the efficiency of autotransplantation surgery.

The success rate was 87.5%, and the 1-year survival rate was 100% in this study. However, the study sample included only eight patients, the follow-up duration was not long enough, and this was not a randomized controlled study; hence, we were not able to substantially conclude whether or not application of computer-aided technique improved the success rate of autotransplantation of teeth (Verweij et al., 2017). Additional studies should be conducted in future.

## Conclusion

In summary, this study described a novel clinical approach for computer-aided autotransplantation of teeth and illustrated the feasibility of this new method, which achieved satisfactory accuracy and stability. This approach facilitated the surgical procedure and might be a viable and predictable method for autotransplantation of teeth.

## Supplemental Information

10.7717/peerj.5939/supp-1 Supplemental Information 1 raw data.

## Additional Information and Declarations

### Data Availability

The following information was supplied regarding data availability: The raw measurements are available in the Supplemental Files.
